# Adaptation of an IRAM W-Band SIS Receiver to the INAF Sardinia Radio Telescope: A Feasibility Study and Preliminary Tests

**DOI:** 10.3390/s23177414

**Published:** 2023-08-25

**Authors:** Adelaide Ladu, Luca Schirru, Pierluigi Ortu, Andrea Saba, Mauro Pili, Alessandro Navarrini, Francesco Gaudiomonte, Pasqualino Marongiu, Tonino Pisanu

**Affiliations:** 1 National Institute for Astrophysics (INAF), Cagliari Astronomical Observatory, Via della Scienza 5, 09047 Selargius, Italy; luca.schirru@inaf.it (L.S.); pierluigi.ortu@inaf.it (P.O.); mauro.pili@inaf.it (M.P.); alessandro.navarrini@inaf.it (A.N.); francesco.gaudiomonte@inaf.it (F.G.); pasqualino.marongiu@inaf.it (P.M.); tonino.pisanu@inaf.it (T.P.); 2 Italian Space Agency, Via del Politecnico, 09133 Rome, Italy; andrea.saba@asi.it; 3 National Radio Astronomy Observatory (NRAO), 1180 Boxwood Estate Rd., Charlottesville, VA 22903, USA

**Keywords:** radio astronomy cryogenic receiver, W-band, Sardinia Radio Telescope, SIS mixer

## Abstract

Radio telescopes are used by astronomers to observe the naturally occurring radio waves generated by planets, interstellar molecular clouds, galaxies, and other cosmic objects. These telescopes are equipped with radio receivers that cover a portion of the radio frequency (RF) and millimetre-wave spectra. The Sardinia Radio Telescope (SRT) is an Italian instrument designed to operate between 300 MHz and 116 GHz. Currently, the SRT maximum observational frequency is 26.5 GHz. A feasibility study and preliminary tests were performed with the goal of equipping the SRT with a W-band (84–116 GHz) mono-feed radio receiver, whose results are presented in this paper. In particular, we describe the adaptation to the SRT of an 84–116 GHz cryogenic receiver developed by the Institute de Radio Astronomie Millimétrique (IRAM) for the Plateau de Bure Interferometer (PdBI) antennas. The receiver was upgraded by INAF with a new electronic control system for the remote control from the SRT control room, with a new local oscillator (LO), and with a new refrigeration system. Our feasibility study includes the design of new receiver optics. The single side band (SSB) receiver noise temperature measured in the laboratory, Trec ≈ 66 K at 86 GHz, is considered sufficiently low to carry out the characterisation of the SRT active surface and metrology system in the 3 mm band.

## 1. Introduction

The 3 mm atmospheric window accessible for radio astronomy observation from the Sardinia Radio Telescope (SRT) site (Sardinia, Italy), approximately 70–116 GHz [1,2], encompasses the standard W-band, 75–110 GHz [3] (wavelength 2.4–4 mm). The design and development of sensitive receivers for the 3 mm band for radio telescopes is relevant to galactic and extra-galactic radio astronomy research. In particular, in our galaxy, observations of small-scale structures in cold molecular gas clouds are performed to search for new molecules and to detect discs around young stars [4], while in nearby galaxies, scientific measurements can be conducted to detect molecules, probe the cold interstellar medium, and study stars’ birth [5,6].

Globally, several radio telescopes are equipped with W-band receivers that enable their scientific research. In particular, the Robert C. Byrd Green Bank Telescope (GBT) [7], located in Green Bank (West Virginia, United States) and with a diameter of 100 m, is equipped with three different receivers that cover all of the frequencies of the W-band (i.e., the W-band 4 mm heterodyne receiver covering 67–93 GHz, the MUSTANG2 bolometric receiver at 80–100 GHz, and the Argus heterodyne receiver covering 74–116 GHz) [8]. In Chile, the Atacama Large Millimeter/Submillimeter Array (ALMA) [9], the world’s largest observatory for millimetre and sub-millimetre waves (35–950 GHz), has been delivering ground-breaking scientific discoveries since 2011. ALMA is an interferometer that operates as a single telescope. It is composed of 66 antennas that can be spread over distances of up to 16 km [10]. Each antenna is equipped with a front end that can incorporate up to ten independent receiver cartridges, which altogether cover the frequency spectrum between 35 GHz and 950 GHz [11]. The 3 mm band is covered by the Band 3 receiver cartridge (i.e., 84–116 GHz) [12]. A new ALMA wideband receiver cartridge, Band 2, to be deployed in the near future, has been designed to cover the 67–116 GHz band [13].

In Europe, another interferometer located on a high mountain in France is available from IRAM (Institute de Radio Astronomie Millimétrique) for high-frequency observations. It was named Plateau de Bure Interferometer (PdBI), and consisted of six antennas operating in the bandwidths 81–115 GHz and 205–245 GHz [14], until it was upgraded and renamed NOEMA (Northern Extended Millimetre Array), currently consisting of the 12-antenna interferometer and equipped with new-generation receivers [15,16].

Concerning the Italian scenario, the 64 m diameter SRT represents the main telescope with which high-frequency observations are possible in conjunction with the Medicina and Noto 32 m telescopes [17]. The SRT is the result of a scientific and technical project carried out by the Italian National Institute for Astrophysics (INAF) and it is managed by the Astronomical Observatory of Cagliari. Although the telescope was designed to operate in a frequency range between 300 MHz and 116 GHz, at present, its maximum observational frequency is 26.5 GHz. In fact, the SRT is currently equipped with three cryogenic front ends, covering a portion of the P-band (305–410 MHz), L-band (1300–1800 MHz) [18], C-band (5.7–7.7 GHz) [19], and K-band (18–26.5 GHz) [20]. The availability of these radio receivers [21] allowed the SRT to be employed in several scientific and space surveillance observations [22,23,24,25]. One of the main technical features of the telescope is its 64 m primary mirror, which is composed of about a thousand aluminium panels sustained by precision mechanical actuators that are digitally controlled to compensate for the gravitational and systematic deformations of the reflector. This feature is known as active surface and it allows an antenna efficiency of up to approximately 40% to be obtained at high frequencies [26].

Some years ago, with the idea of gaining experience with a radio receiver that operates at high frequencies and testing the performance of the SRT (i.e., telescope active surface and metrology system), the Astronomical Observatory of Cagliari acquired a dismissed W-band receiver developed by IRAM, which was installed on one of the PdBI antennas until 2006. The W-band receiver prototype from IRAM, described in this paper, was adapted and characterised in the INAF laboratory. However, the installation of the prototype in the SRT antenna was not finalised, since in the meantime, the Italian Ministry of University and Research funded the major INAF proposal for the SRT high-frequency upgrade [27], known as the National Operational Program grant (PON), which included the development of a high-performance W-band multi-beam receiver in the Work Package n. 1, as known as PON OR1. The commissioning of the novel instrumentation acquired for the SRT in the framework of the PON grant is underway and the results will be described in separate articles.

The receiver acquired from IRAM is a dual-feed system that covers two frequency bands simultaneously: a portion of the W-band (84–116 GHz), also known as the 3 mm band, and a frequency range between 210 and 248 GHz (1.3 mm band) [28,29]. The instrument observed single linear polarisations and operated at cryogenic temperature to achieve a low receiver noise temperature [28,29]. All of the components of the receiver could be controlled and managed by a microcontroller using an Ethernet link.

Since the SRT was designed to cover all frequencies up to 116 GHz, only the 3 mm channel of the ex-PdBI receiver was upgraded and is presented in this work. The original version of the instrument was unsuitable for the SRT and it required significant modifications and refurbishment from the point of view of its structural and logistical characteristics and its electronic control system [30,31].

In this paper, a feasibility study of the upgrade of the ex-PdBI receiver for the adaptation to the SRT is presented. One of the goals of the upgrade was to equip the receiver with a new electronic control system based on onboard intelligence that permits smart remote control. In Section 2, we present the original version of the receiver, and focus on its signal acquisition chain of the 3 mm channel. In Section 3, all structural and logistical adaptations for the installation on the SRT are reported (i.e., the feasibility study of the receiver optics, the upgrade of the refrigeration system, and the evaluation of a new local oscillator) are reported. The new electronic control system, with the onboard intelligence to remotely control the receiver from the control room of the SRT, is described in Section 4. Finally, the results and discussion about the receiver performances with these new modifications are presented in Section 5.

## 2. Materials and Methods: The Original Version of the Ex-PdBI W-Band Receiver

**Figure 1:**
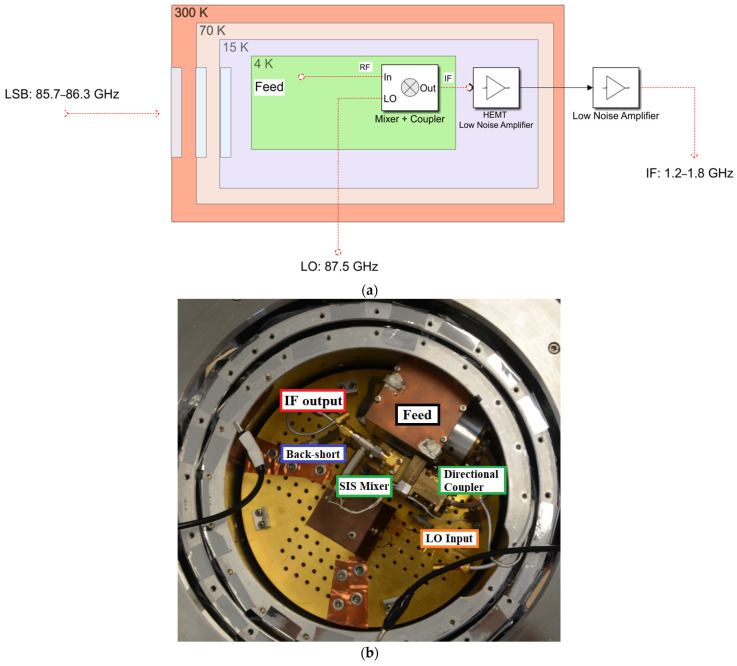
(**a**) Schematic of the signal acquisition chain of millimetre-wave and RF components of the original version of the ex-PdBI receiver. The mixer and the LO coupler are thermally cooled at 4 K inside a cryostat with 70 K, 15 K, and 4 K cryogenic stages. (**b**) Photo of the signal acquisition chain in the Dewar.

The ex-PdBI receiver consists of a single cryogenically cooled cryostat equipped with two frequency channels: one for the 84–116 GHz band and the other for the 210–248 GHz band [28]. Since the SRT was designed to cover all frequencies up to 116 GHz, only the 84–116 GHz channel is considered. The original version of the system permits the detection of only linearly polarised signals. Figure 1a shows a schematic of the original version of the receiver.

The feed system consists of a circular corrugated horn that has a flare angle of 7.42 degrees, an aperture diameter of 12.4 mm, and a length of 39.2 mm [28,29]. A phase error at the aperture of approximately one-tenth of the wavelength (i.e., approximately 3 mm) and a beam waist close to the aperture is consequently generated by the feed. A rectangular-to-circular guide step transformer is installed downstream from the feed horn. An optical system composed of a high-density polyethylene (HDPE) lens is designed and placed at the aperture of the horn to compensate for the phase error and to optimally position the beam waist. Owing to this form of optics, the antenna beam for the receiver is adapted for the optics of the PdBI telescopes, guaranteeing a sub-reflector edge taper of approximately −12 dB with an aperture angle of ±5.6 degrees. Further details about the HDPE optical system and the feed system are reported in [28,29]. A polarisation grid is installed on the receiver to allow only one linear polarisation (i.e., the horizontal or vertical contribution) to enter the feed, depending on the orientation of its wires. In this manner, the receiver provides a linearly polarised electromagnetic signal at the back end.

**Figure 2:**
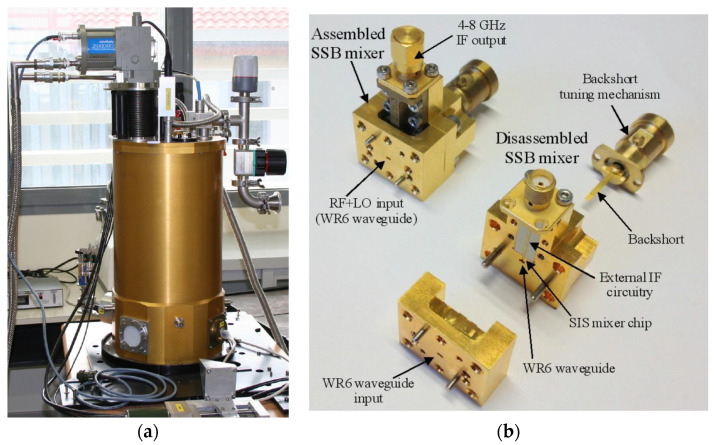
(**a**) Photo of the ex-PdBI receiver mounted on a laboratory test bench. (**b**) Photo of the single side band (SSB) superconductor–insulator–superconductor (SIS) mixer.

The ex-PdBI receiver utilises a down-conversion system, with a mixer as the first active element of the chain. The weak 3 mm wave signal ν_RF_ from the radio astronomy source (in the 84–116 GHz range) is added to a relatively strong local oscillator (LO) monochromatic signal ν_LO_ produced locally and coupled into a non-linear element, a superconductor–insulator–superconductor (SIS) mixer (see Figure 1a,b). The output signal at the frequency difference ν_IF_ =|ν_LO_ − ν_RF_|, named the intermediate frequency (IF), preserves the amplitude and phase of the original RF signal. The down-converted IF signal falls in the 1.2–1.8 GHz range. This IF range falls within the 0.1–2.1 GHz baseband of the SRT. The down-conversion operation is carried out using a backshort-tuned single side band (SSB) SIS mixer similar to the one illustrated in Figure 2b [32,33], which operates at the cryogenic temperature of 4 K. The SSB SIS mixer design includes a rectangular waveguide with a single adjustable non-contacting backshort for the tuning, which achieves a good matching of the SIS junction impedance in the signal band (typically lower side band, LSB, ν_LSB_ = ν_LO_ − ν_IF_) in any portion of the 84–116 GHz receiver band, while rejecting the image sideband (typically upper side band, USB, ν_USB_ = ν_LO_ + ν_IF_). The achieved image sideband rejection is about 15 dB at the centre of the IF band (around 1.5 GHz). The SIS junction consists of two layers of superconducting metal (niobium) separated by a thin insulator layer of aluminium oxide a few nanometres thick. Typically, the SIS junctions have a size of the order of a few μm^2^ and their integrated thin-film superconducting tuning circuitry sits on top of a quartz substrate, which is itself mounted across a waveguide [31].

Regarding the local oscillator, the receiver was developed to use the Gunn-based local oscillator from IRAM telescope, which exploits the performances of a semiconductor device (Gunn diode) to achieve oscillation at a precise frequency [34]. A side-wall coupler, with a coupling of about 20 dB and an insertion loss of about 0.2 dB, is used to inject the local oscillator into the signal path [28]. The coupler is connected to the horn using a brass bracket, which also acts as a support for the mixer and flange between the mixer and coupler.

The IF output from the SIS mixer is connected to an isolator (see Figure 1b), with a stainless-steel coaxial cable. The down-converted IF signal is amplified by a low noise amplifier (LNA) [35] that delivers an instantaneous bandwidth of 600 MHz, based on high-electron-mobility transistor (HEMT) technology, with a gain of about 30 dB and a noise temperature of about 5 K.

All of these components are installed on a hybrid cryostat (HDV10 from Infrared Laboratories) [36], or Dewar (see Figure 1b and Figure 2a), in which there are three cryogenic stages: two of them (i.e., 70 K and 20 K) are achieved thanks to a CTI model 350CP cold head [37] and the other (i.e., 4 K) is provided by a 10-inch-diameter high thermal conductivity plate in thermal contact with a vessel of liquid helium. The mixer, local oscillator coupler, circular horn, polarising grid, and optical lenses are mounted on the 4 K stage. The isolator and the LNA are mounted on the 20 K screen, connected to a stainless-steel inner and outer conductor coaxial cable. The SSB SIS receiver, including the SIS mixer, the IF LNA, the feed, and optics, deliver a low receiver noise temperature of less than 50 K when measured in front of the cryostat vacuum window.

The receiver chain in the Dewar is followed by a chain section operating at room temperature. It provides another amplification stage, with a gain of about 60 dB, and a programmable step attenuator from 0 to 20 dB, which is useful for controlling the amplitude level of the signals that are fed into the back end for sampling and later processing.

The noise temperature of radio astronomy receivers is one of the most important performance parameters that directly impact the sensitivity achieved during radio astronomy observation. The ex-PdBI W-band receiver is equipped with a classical calibration system that performs noise temperature measurements using the cold and ambient loads method. The noise temperature of the receiver is evaluated at its input vacuum window and positioned on the surface of the Dewar. The receiver calibration system is composed of two loads at room temperature and cryogenic temperature, respectively. The load at room temperature is an absorbing panel of a squared shape made of Emerson & Cuming AN72 material [38], which can be positioned in the receiver signal path with a dedicated direct current (DC) motor. The cold load consists of an absorbing panel suited to use at 4 K, positioned into the Dewar, which can be activated by moving a rooftop mirror with normal angle of reflection, located on the outside of the cryostat at room temperature. The rooftop mirror can be moved along the receiver signal path using a dedicated DC motor. This rooftop mirror allows the rotation of the polarisation angle of the incoming beam by 90 degrees and the reversal of its direction towards the cryogenic load.

The original receiver control system permits the control of all receiver functions through a VME-based microprocessor. All communications with the control computer are possible thanks to an Ethernet link.

## 3. Structural and Logistical Adaptation of the Ex-PdBI W-Band Receiver for the Installation on the Sardinia Radio Telescope

As mentioned above, the receiver was originally designed to be installed on a PdBI antenna. Consequently, a feasibility study for some structural and logistical modifications is needed with a focus on adapting the system to the optics and mechanical constraints of the SRT. In particular, a feasibility study based on the optical design of the receiver is necessary to demonstrate that it can be installed on the SRT.

The optical design of the SRT is based on a quasi-Gregorian configuration with a shaped primary mirror (i.e., 64 m diameter) and a shaped sub-reflector (i.e., 7.9 m diameter) that make available two focal positions (i.e., the primary focus, with a focal length to diameter ratio equal to 0.33 and frequency range 0.3–20 GHz, and the Gregorian focus, with a focal length-to-diameter ratio equal to 2.34 and frequency range 7.5–116 GHz). In addition, the SRT has a beam wave guide (BWG) room, where four positions are available for 2.9 m mirrors and one mirror with a diameter of 3.9 m, for a total of four additional focal positions (i.e., the F3 and F4 focus, with a focal length-to-diameter ratio equal to 1.38, and the F5 and F6 focus, with a focal length-to-diameter ratio equal to 2.81 and frequency range 1.4–35 GHz).

The ex-PdBI W-band receiver can be installed on the Gregorian focus of the telescope. An octagonal support structure, located in the Gregorian room surrounding the SRT Gregorian focus, can host up to eight receivers. This structure, named a Gregorian receiver positioner (GRP), rotates around an axis parallel to the antenna optical axis allowing the placement of the selected receiver on the precise point (the Gregorian focus) for astronomical observation. Since the Dewar of the receiver is equipped with an inner vessel of liquid helium to achieve a temperature of 4 K, a new cooling system was necessary to prevent the leakage of the cryogenic fluid when the telescope moves in elevation. In addition, a new local oscillator, which replaces the traditional system based on Gunn oscillators installed on the PdBI antennas, was adopted.

The feasibility study to demonstrate that the ex-PdBI receiver is suitable for the Gregorian focus of the SRT is presented in Section 3.1. In Section 3.2, the features of the new local oscillator for the SRT are reported. The new cooling system, based on a new cold head with high performance to replace the old system, is described in Section 3.3.

### 3.1. Feasibility Study for the Installation on the Gregorian Focus of SRT

As mentioned above, the ex-PdBI receiver can be installed on the Gregorian focus of the SRT, which offers a focal length-to-diameter ratio equal to 2.34 and can host radio receivers that work in the frequency range of 7.5–116 GHz. At this focal position, the angle subtended by the edge of the sub-reflector as seen from the optical axis is about 12 degrees. The optimum edge taper, the ratio of power at the edge of the SRT sub-reflector with respect to the on-axis value, is approximately 13 dB.

Some feasibility studies were conducted and the receiver with its original cooling system (based on the helium vessel) and with the upgraded version (explained in the following Section 3.3) were both considered. In [30,31], the results of these studies are reported in detail.

As described in Section 2, the HPDE optical system of the receiver matched with its feed horn produces a beam waist that meets the edge taper requirements of the Gregorian focus of the SRT. As a final result, the chosen configuration of installation (presented in Section 3.3) permits a perfect match between the output beam waist of the receiver and the beam waist of the SRT.

### 3.2. Need for a New Dedicated Local Oscillator: The ALMA Band 3 Local Oscillator

As previously described, the W-band receiver builds on a down-conversion system that uses an SIS mixer, which was originally pumped with a traditional Gunn diode located at room temperature outside the cryostat. For the installation on the SRT, a new dedicated local oscillator that operates at high frequencies, also placed at room temperature, was adopted. This local oscillator was developed by the National Radio Astronomy Observatory (NRAO) for the ALMA Band 3 (84–116 GHz) project [39]. It was expected to meet the main requirements of frequency range tuneability and delivered output power. The ALMA Band 3 LO module consists of an active multiplier chain (AMC) in cascade with a power amplifier (PA). In our arrangement for the SRT, we injected a signal at the input of the AMC from an external signal generator (Rohde and Schwarz signal generator, model SMF 100A [40]). The generator synthesises a single-tone continuous signal tuneable across the 15.333–18.000 GHz frequency range, with a power level between 10 dBm and 15 dBm (in the ALMA system, the 15.333–18.000 GHz signal is provided by a commercial YIG oscillator). In the AMC, this signal is frequency multiplied by 6 (first by 2, then by 3), filtered, and amplified in order to achieve the desired W-band local oscillator frequency (i.e., 92–108 GHz) with an output power level in the range 1–10 mW. The AMC module incorporates two monolithic microwave integrated circuit (MMIC) multipliers (double and triple multipliers) and an MMIC W-band amplifier. Since the ALMA receivers are equipped with two orthogonal polarisation channels, the PA module splits the signal from the AMC module in two and further amplifies these new signals thanks to a user-controlled amplitude system, with a step of 0.2 dB, in order to achieve a power value between −4 dBm and 2 dBm. Only one of the two PA output channels is used for the ex-PdBI receiver, whereas the other channel is terminated to 50 Ohm load. The LO output of the PA is available from a standard WR10 waveguide with UG385 flange. A section of interconnecting WR10 waveguide must be used between the PA and the WR10 waveguide vacuum feedthrough located at the LO input interface of the receiver cryostat.

**Figure 3:**
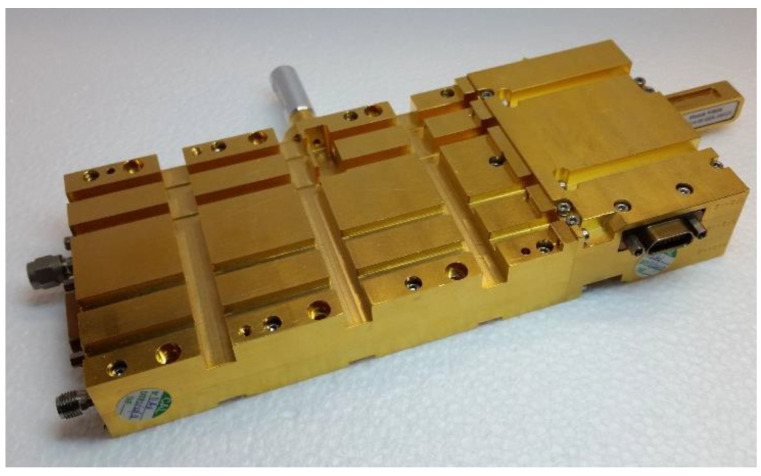
Photo of the ALMA Band 3 local oscillator showing the AMC (left module) and PA (right module) connected to each other.

The W-band receiver bandwidth 84–116 GHz is the same as that of the ALMA Band 3. However, the IF range of the ex-PdBI receiver falls between 1.25 and 1.75 GHz (500 MHz bandwidth), as opposed to the 4–8 GHz bandwidth of the ALMA Band 3 [39]. Consequently, the local oscillator bandwidth required to cover the desired radio frequency band for SRT is 85.75–114.25 GHz, which is larger than the bandwidth of the ALMA Band 3. Some preliminary tests were necessary to test the ALMA Band 3 local oscillator in the extended frequency range of the ex-PdBI receiver with the goal of deriving the optimal power level for achieving high performances. Figure 3 shows a photo of the ALMA Band 3 local oscillator. The results of these tests are presented in Section 5.

### 3.3. New Cooling System Based on the ARS Cold Head, Model DE-204SF, to Achieve the Temperature of 4 K

**Figure 4:**
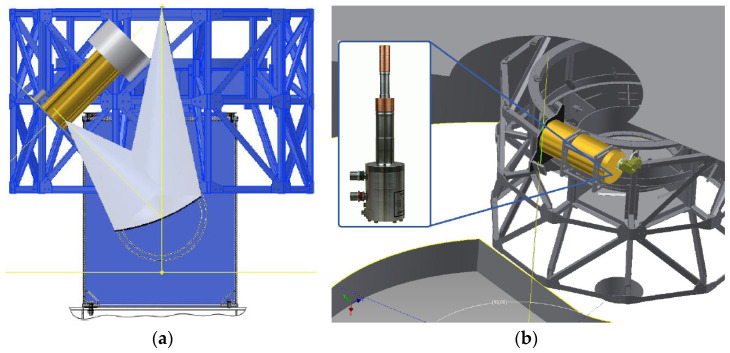
(**a**) Sketch of the tilting installation, where the Dewar is placed at an angle of 45 degrees from the zenith direction on the Gregorian receiver positioner (GRP). (**b**) Sketch of the horizontal installation with the new cryogenic cooler.

Unfortunately, the architecture of the ex-PdBI receiver is constrained by its cryostat. As mentioned above, the receiver is based on a hybrid Dewar from Infrared Laboratories (i.e., model HDV10 [36]) with two cryogenic stages (i.e., 80 K and 15 K) provided by a commercial closed-cycle cold head from the CTI-Cryodine, model 350 CP [37], and a 4.2 K stage provided by a 10-inch diameter high thermal conductivity plate in thermal contact with an inner vessel of liquid helium (7 L in size). To avoid spilling out this cryogenic fluid when the telescope moves in elevation, the Dewar could be placed at an angle of 45 degrees from the zenith direction (see Figure 4a). In this configuration, regardless of the elevation-pointing angle, the receiver will always be within ±45 degrees from the vertical axis of the telescope. For example, the receiver is oriented at −45 degrees when the telescope points towards the horizon (i.e., elevation pointing of 5 degrees). Unfortunately, some design and logistical problems rule out the possibility of applying this solution. For example, the amount of liquid helium in the inner vessel decreased to 5 L in this configuration of the tilted installation. However, the availability of 5 L of liquid helium ensures that the 4.2 K temperature holds for more than 15 days before a new refilling is required. A notable limitation of this tilting installation is that switching to other receivers, installed in the support structure of the Gregorian room, is impossible until the liquid helium of the vessel receiver is fully evaporated. In fact, when putting into the Gregorian focus on another receiver, the Dewar is positioned along the horizontal direction for some telescope pointing angles and helium could leak from the cryostat. This aspect restricts the potential of SRT, which was designed for frequency agility, i.e., the possibility to observe the sky at several frequencies (i.e., from 0.3 to 116 GHz) by an easy a quick switch of between different receivers. The main restriction is that there is not enough space in the Gregorian room for the implementation of the tilting solution. In the tilting solution, because the feed is placed on the lower part of the receiver and this is rotated at −45 degrees from the vertical axis, a new elliptical mirror external to the receiver is required to orient the beam from SRT to the feed. Unfortunately, the total size of this system would exceed the available space.

An improved solution based on an additional close-cycle cryogenic cooler was proposed where the temperature of the third cryogenic stage of the receiver (i.e., 4 K) can be achieved by excluding the original refrigeration system based on liquid helium. This new cryogenic cooler that we adopted replaces the previous CTI 350CP cold head and allows the antenna to be pointed in any direction of the sky without causing problems to the receiver functioning. The chosen cold head, from Advanced Research Systems (ARS), model DE-204SF [41], is characterised by two cryogenic stages (i.e., 20 K and 4 K) and provides 8 W and 0.2 W of cooling power, respectively.

A mechanical modification of the Dewar was required to host the new cold head. By adopting this solution, it would be possible to place the receiver horizontally in the upper part of the rotating turret, in correspondence with the Gregorian focus (see Figure 4b), such that the output beam waist of the external elliptical mirror and the beam waist of the SRT are matched.

## 4. New Electronic Control System of the Receiver for the Installation on SRT

The original control system of the ex-PdBI receiver consists of three control boards developed by IRAM, two of which are used to control the SIS mixer (and the Gunn-based local oscillator) and the other being dedicated to the management of the calibration system. All boards are connected to a VME-based microprocessor using an Ethernet link. These control boards are devoid of on-board intelligence and they are unable to operate in a telescope such as the SRT, where the remote control of the receiver is essential. Therefore, a new ad hoc electronic control system has been designed and developed to make the receiver suitable for the SRT.

**Figure 5:**
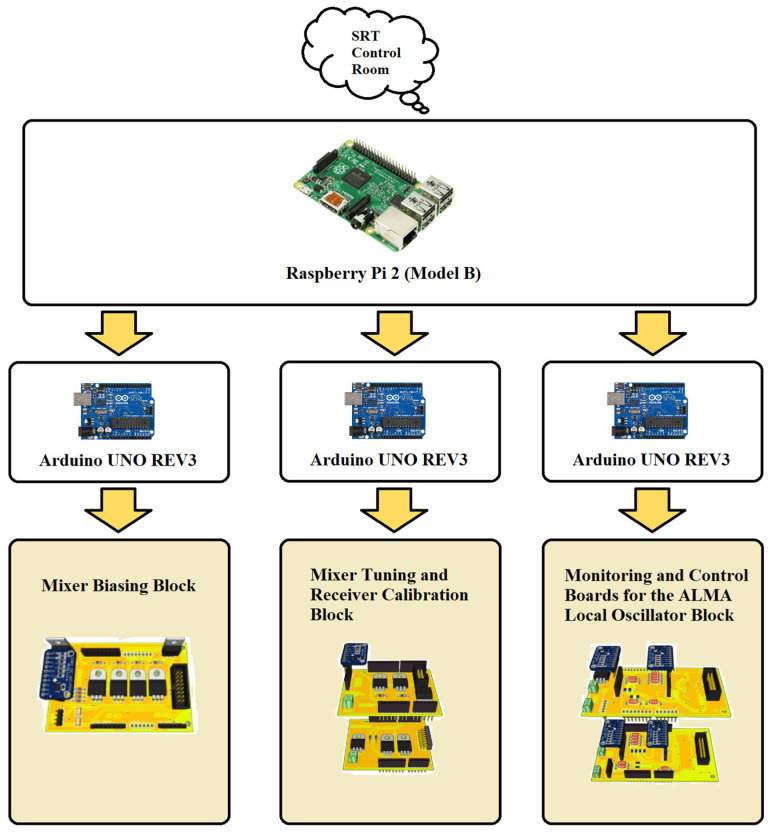
Architecture of the new electronic control system.

The new electronic control system is composed of a Raspberry Pi 2 (Model B) [42], which serves as the brain of the system and controls three different Arduino UNO REV3 microcontrollers, based on Atmel ATmega 328P [43]. Each Arduino UNO REV3 microcontroller manages one or more electronic boards dedicated to a specific function for the receiver operation: biasing the SIS mixer, controlling the receiver calibration system, and biasing and tuning the ALMA local oscillator. The Raspberry Pi 2 board permits linking of the receiver to the SRT software control and managing and it consequently makes remote control of the receiver available. The architecture of the electronic control system is illustrated in Figure 5. Table 1 summarises the technical features and functioning of each subsystem.

All components of the new control system are integrated into a unique rack, which can be installed close to the cryostat. With the aim of reducing the self-generation of radio frequency signals, which represents a noise for the radio astronomy observations performed with others SRT receivers (i.e., P, L, C, and K frequency bands [18,19,20]), all electronic boards are enclosed in aluminium boxes.

### 4.1. Raspberry Pi 2 (Model B): The Core of the Electronic Control System

Before astronomy observations can be performed with the ex-PdBI W-band receiver, some settings need to be executed to prepare the instrument properly. These settings must be executed remotely from the SRT control room by the astronomers that use the telescope.

In particular, tuning the ALMA local oscillator and locking it to the desired frequency is required for each observation. To do this, the backshort of the SIS mixer is placed at the proper position for the chosen frequency, and the junction bias voltage is set. The local oscillator power supply is adjusted to achieve a junction direct current of the order of 20 μA [39]. Typically, this set of setting operations takes a few minutes and it should be completely transparent to the astronomers that use SRT to collect data for scientific research. This is possible because of the single-board computer Raspberry Pi 2 (Model B), which is programmed with ad hoc Python scripts [31], which represents the core of the new control system and allows the remote control of the receiver. The Raspberry Pi 2 is chosen for its quad-core processor, which runs at 900 MHz with 1 gigabyte of random-access memory (RAM) for its USB and Ethernet ports (i.e., four and one, respectively) and because it made it possible to use an operative system based on Linux open-source software [42], as well as its small size.

Apart from the technical features of the device that are perfect for the requirements to be met, the self-generation of radio frequency interference (RFI) represents a critical issue for a system that will be installed on a radio telescope. In this respect, an accurate measurement campaign was conducted with the aim to obtain knowledge of all self-produced signals and validate the logistical solutions for their mitigation. Since SRT was designed to operate from 300 MHz to 116 GHz, and considering that some receivers up to 26.5 GHz are installed on it, the RFI measurement campaign is performed in the frequency range of 0.1–6 GHz. The measurement campaign is performed in free space using a log-periodic antenna, which operates in the frequency range between 250 MHz and 7 GHz, and the Rohde and Schwarz FSV40 Spectrum Analyzer as the back end [31]. Three different types of measurement are carried out:

- Raspberry powered off, to highlight all signals coming from known external sources (i.e., frequency modulation radio signals, mobile communications signals, etc.) and to establish a reference level baseline;
- Raspberry powered on without a shield to highlight all signals that are self-generated by the internal clock and Ethernet technology of the device;
- Raspberry powered on with a double aluminium box to highlight the strong attenuation of the signals that are self-generated by the device.

The results of the RFI measurement campaign are reported in [31]. There was evidence that positioning the Raspberry in a double aluminium box resulted in almost sufficient attenuation of the self-produced signals. The signals across the P-band (i.e., 300–400 MHz [18]) are the only ones that have a relevant amplitude. In the case of P-band observations with SRT, the W-band receiver control system can be turned off.

For the monitoring and control of the system, a dedicated software (named Tester100GHz) based on Microsoft Visual Basic .NET has been developed to allow communication with the Raspberry module. This sends and receives messages from the three satellite cards. The Tester100GHz uses a graphical user interface (GUI) that allows the user to configure the system in a simplified manner.

### 4.2. Mixer Biasing Block: The SIS Mixer Biasing Control Board

**Figure 6:**
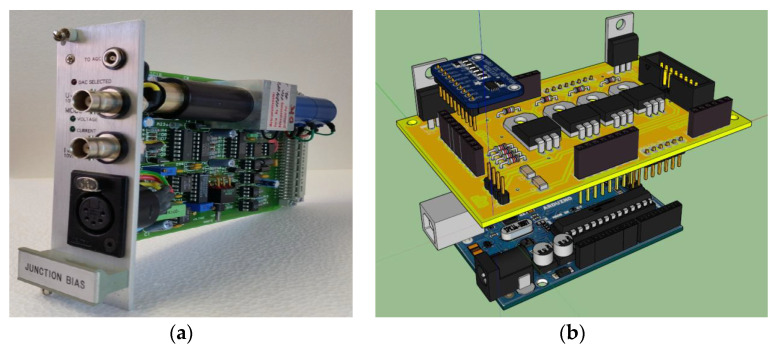
(**a**) SIS mixer junction bias module developed at IRAM. (**b**) Three-dimensional rendering of the SIS mixer biasing control board developed for SRT.

One side of the SIS junction is connected to a properly designed interconnecting bias circuitry that acts as an interface with the outside. This circuitry is implemented by IRAM in the so-called Junction Bias (JB) board (see Figure 6a), which permits both bringing out the IF signal and to providing bias driving in voltage or current modes [31]. This board, equipped with an internal reference voltage, provides the input junction voltage or current using a DC motor with a potentiometer or a digital-to-analogue converter (DAC), respectively. To evaluate the optimal biasing of the SIS junction, the board is fitted with two analogical outputs that trace the I/V characteristics. Unfortunately, the IRAM JB board has no on-board intelligence and is unsuitable for a receiver that is installed on the SRT. For these reasons, a new electronic control board has been developed for the purpose of managing the IRAM JB board (see Figure 6b), remotely. The new control board is equipped with a power supply stage designed to provide different voltage supplies (i.e., 5 V for digital logic circuit and for the potentiometer calibration, ±15 V for operational amplifiers, and 12 V for motor–potentiometer), a readout interface, and a control stage of the potentiometer motor. The readout stage is based on a high-resolution analogue-to-digital converter (ADC), model ADS1115, fitted on Adafruit modules from Texas Instruments [44] that allows the reading of the voltage and current values about the junction biasing, the potentiometer motor position, and the DAC of the IRAM JB board. The control stage of the potentiometer motor enables the tuning of the potentiometer using digital controls to set a specific current or voltage value for the SIS mixer junction. Furthermore, this stage also permits the setting of supplies for both the motor–potentiometer configuration and the DAC configuration of the IRAM JB board.

A dedicated module of Tester100GHz reads all ADC values and set the voltage values managing the potentiometer or the DAC.

### 4.3. Mixer Tuning and Receiver Calibration Block

**Figure 7:**
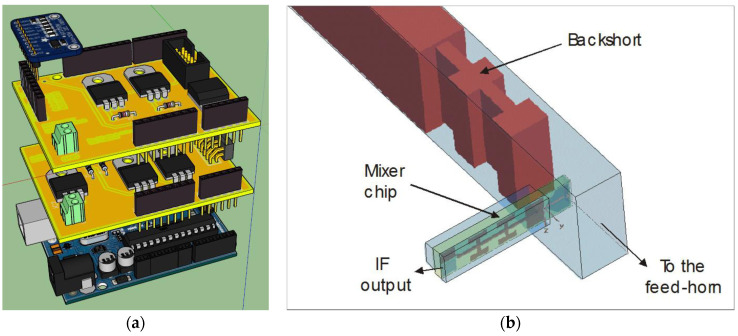
(**a**) Mixer tuning and receiver calibration block, composed of the SIS mixer backshort motor control board and the receiver calibration motor control board. (**b**) Inner waveguide structure of the SIS mixer showing the movable backshort and the mixer chip [32,33].

The mixer tuning and receiver calibration block is composed of two electronic boards, both controlled by a single Arduino UNO REV3 microcontroller. Only one microcontroller is enough to handle the signals and functions of these two electronic devices. These boards are fundamental to calibrate the ex-PdBI receiver, evaluating its noise temperature, and for tuning its operating frequency. In particular, these two electronic devices are (see Figure 7a):

- The SIS mixer backshort motor control board;
- The receiver calibration motor control board.

**Figure 8:**
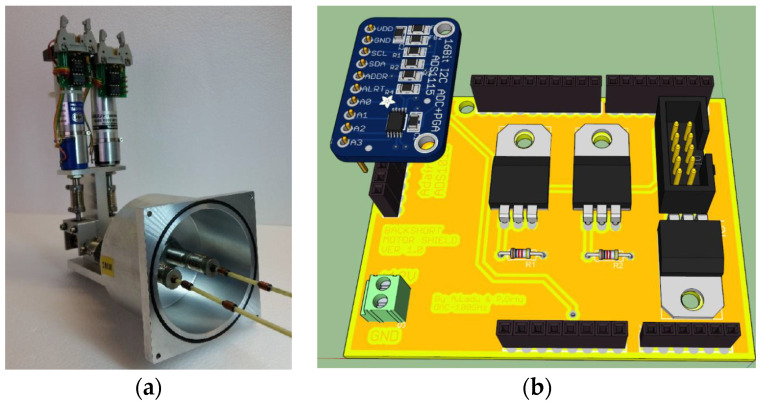
(**a**) IRAM backshort system. (**b**) three-dimensional rendering of the SIS mixer backshort motor control board developed for SRT.

The embedding impedance the SIS mixer due to its integrated superconducting circuitry in the waveguide structure is frequency-dependent. A suitable mixer tuning is obtained by adjusting the length of the waveguide section between the SIS junction block and the backshort (see Figure 7b). IRAM developed a system, devoid of onboard intelligence, based on a DC motor with a potentiometer for backshort regulation (moving forward or backward) with respect to the junction (see Figure 8a). Highly accurate positioning results can be achieved by supplying short pulses to the motor. This IRAM board was placed at the top of the device to control the motor displacement. A new electronic board has been developed to remotely control the IRAM board (Figure 8b). This new board, named an SIS mixer backshort motor control board, features a power supply block, readout block, and motor control block. The board is managed using a second Arduino UNO REV3 microcontroller [43]. The readout block is equipped with a high-resolution ADC, model ADS1115, fitted on Adafruit modules from Texas Instruments [44], for the evaluation of the backshort motor position based on reading its voltage values. The motor control block allows adjustment of the potentiometer using digital commands to position the backshort of the mixer at the desired position.

As explained in Section 2, the ex-PdBI W-band receiver is equipped with a classical calibration system based on the cold and ambient load method. The cryogenic and room temperature loads are installed on their respective DC motors, which allow their correct positioning for receiver calibration.

**Figure 9:**
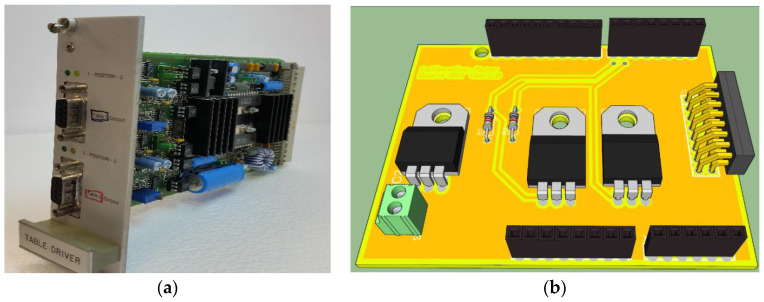
(**a**) IRAM board for the management of the calibration system; (**b**) 3D rendering of the new control board for the calibration motors developed for SRT.

The control system for the management of the receiver calibration block requires switching from two available positions for the DC motor: calibration on and calibration off, respectively. These features are provided by the motor position with respect to two inductive proximity switches: the first for the rest position (i.e., calibration off) and the second one for the working position (i.e., calibration on). IRAM developed an electronic board for the execution of these operations (see Figure 9a) devoid of onboard intelligence, and the remote control is unavailable. Therefore, a new receiver calibration motors control board, equipped with a power supply stage and a motor control stage, was developed (see Figure 9b). The motor control stage permits moving the DC motor between the two working positions (i.e., calibration on and calibration off positions) and collecting information about its state.

A dedicated module of Tester100GHz was developed to control and test the mixer tuning and receiver calibration block. The module is divided into two parts: one for the mixer tuning and the other for the receiver calibration system, respectively. The first one permits reading the ADC data and setting the potentiometer to select the optimal voltage. This value of voltage can be continuously monitored by the user. Instead, the part of the software for the calibration system enables the DC motor’s movement thanks to the availability of two side bars, and the monitoring of their status, with specific indicators for any anomalies.

### 4.4. Monitoring and Control Boards for the ALMA Local Oscillator at SRT

As mentioned above, the adaptation of the ex-PdBI receiver to SRT involves the installation of the NRAO local oscillator developed for the ALMA Band 3 project. A new ad hoc prototype of an electronic board has been developed to remotely tune this local oscillator. This prototype is composed of two modules: one for managing the AMC and the other for controlling the PA, respectively. Since the two modules require high DC power supplies (i.e., AMC/PA: +6 V and 1.5 A, ±15 V and 500 mA), each control board is equipped with a power supply stage that ensures high stability at high currents. In addition, a further protection stage to prevent malfunction is built on each board.

**Figure 10:**
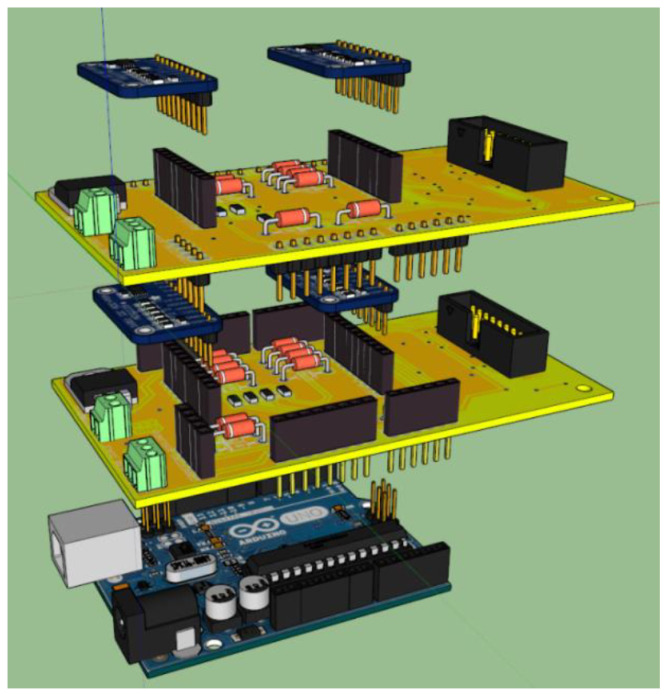
Ad hoc electronic board for the control of the AMC and PA modules of the ALMA local oscillator.

The board for the control of the AMC module is designed to provide feedback for the voltages by the power supply stage. The board is based on two high-resolution ADCs, model ADS1115, fitted on Adafruit modules from Texas Instruments [44], which are capable of reading all AMC voltages with 16-bit resolution. The board is equipped with an inter-integrated circuit (I2C) bus for communication with the Arduino UNO REV3 microcontroller (see Figure 10).

The PA module is controlled such that the gate and drain voltages can be set independently. The gate and drain voltages are set by two programmable potentiometers, one for each PA output channel. The remote control of these potentiometers and data sending are possible thanks to a serial peripheral interface (SPI) bus, which is directly connected to the Arduino microcontroller. The PA control board is also equipped with two high-precision ADCs [44] that provide feedback about the state of the amplifier junction’s polarisation.

A dedicate module of Tester100GHz was developed to test the monitoring and control electronics boards for the ALMA Local Oscillator [31], as well as for the other electronic boards. This module permits the reading of all voltage values generated by the two boards and is useful for the tuning of the ALMA local oscillator. The whole system is equipped with an I2C bus, which serves to the communication between the ADCs of the two boards and the Arduino microcontroller, and a serial peripheral interface (SPI) bus to communicate between the potentiometers and the Arduino microcontroller. The module provides real-time feedback to the users.

## 5. Results and Discussion

The ex-PdBI receiver was upgraded for its installation on the SRT. The following modifications were made:

- Its cooling system was redesigned, as described in Section 3.3;
- The ALMA Band 3 local oscillator was used as a replacement for a Gunn oscillator;
- A new electronic control system was developed for the remote control from the control room of SRT (Section 4).

Following these changes, tests and measurements were necessary to evaluate the performance of the system. In Section 5.1, we describe the suitability of the ALMA Band 3 local oscillator, considering the power requirements and extended LO frequency range 85.75–114.25 GHz to drive the ex-PdBI receiver with its optimum parameters. The measurements of the noise temperature in the bandwidth of the receiver are reported in Section 5.2.

### 5.1. Evaluation of the Output Power of the ALMA Band 3 Local Oscillator

The performances of a receiver based on an SIS mixer depend on the pumping levels applied by the local oscillator [45]. Laboratory measurements were performed to study the optimum output power level of the ALMA Band 3 local oscillator to bias the SIS junction of the mixer. A signal generator (model SMF100A from Rohde & Schwarz [40]), a digital oscilloscope (model RTO 1044 from Rohde & Schwarz [40]), and a power meter (model PM5 from Erickson [46]) were used to perform these measurements.

The Raspberry Pi 2 was programmed to enable the control of the laboratory instruments to run the tests. The frequency and amplitude sweep of the signal, synthesised by the signal generator and directly connected to the input of the ALMA Band 3 local oscillator, were varied in pre-established steps. The power meter, connected to the output of the local oscillator, measured the output power levels and saved the data simultaneously. These data were compared with the nominal power levels required for the SIS mixer biasing. The order of magnitude of the LO pumping power level required at the junction, named *P_LO_*, is given by the following formula (we assume to bias the mixer on the first photon step below the gap voltage of the superconductor) [45]:

(1) $$P_{LO}=\frac{{\left(N\cdot h\cdot \nu_{LO}\right)}^{2}}{2\cdot R_{N}\cdot e^{2}}$$

where *N* represents the number of the SIS mixer junctions in series, *h* is the Planck’s constant, *ν_LO_* is the LO frequency, *R_N_* is the normal-state resistance of the current–voltage (I-V) SIS junction characteristic, and *e* the electron charge. The minimum required LO power level at the SIS junctions is of the order of 10 nW at 100 GHz for an SIS mixer with a single junction (*N* = 1) and 40 nW for a two-junction series array (*N* = 2), like the one used in the ex-PdBI 3 mm band SIS mixer. The attenuation of each component between the local oscillator injection coupler and the SIS mixer module is considered for the evaluation of the LO output power levels required for the local oscillator at the WR10 LO waveguide output flange of the cryostat. In detail, the LO coupler module (attached to the SIS mixer module is connected through a waveguide transition (with an insertion loss of approximately 6 dB) to the WR10 waveguide output of the ALMA Band 3 local oscillator (located outside the cryostat). The LO coupler (see Figure 1) has a coupling value of approximately −28 dB. The coupling value is small so as to minimise the noise added to the signal path. The LO output power required at the WR10 output waveguide flange of the PA be more than three orders of magnitude greater than the nominal power level calculated using (1). The minimum value of the required LO power is taken to equal to 2 mW across the 92–108 GHz band.

**Figure 11:**
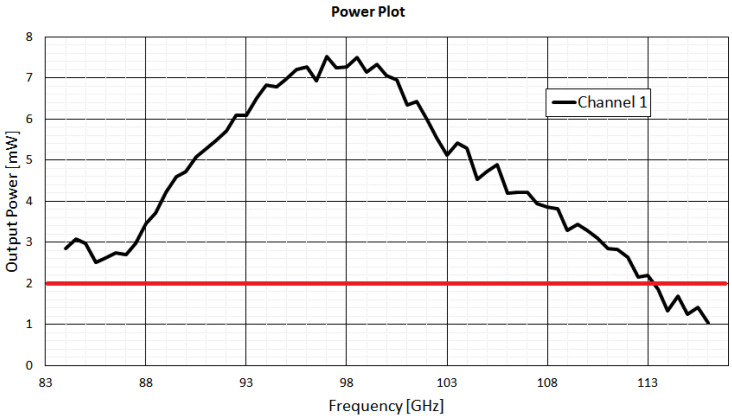
Measured output power from the channel 1 of the ALMA Band 3 local oscillator. In red, the minimum value of 2 mW is highlighted. The drain and gate voltage values are set at 1.67 V and −0.1 V, respectively.

Figure 11 shows the plot of the output power of the local oscillator (channel 1). The drain and gate voltage values are set at 1.67 V and −0.1 V, respectively. The curve shows a peak power of approximately 7 mW at 98 GHz. The minimum value of 2 mW (highlighted in red) is exceeded in almost all local oscillator bandwidths from 85.75 to 114.25 GHz. These requirements can be easily achieved by optimising the drain and gate voltages.

**Figure 12:**
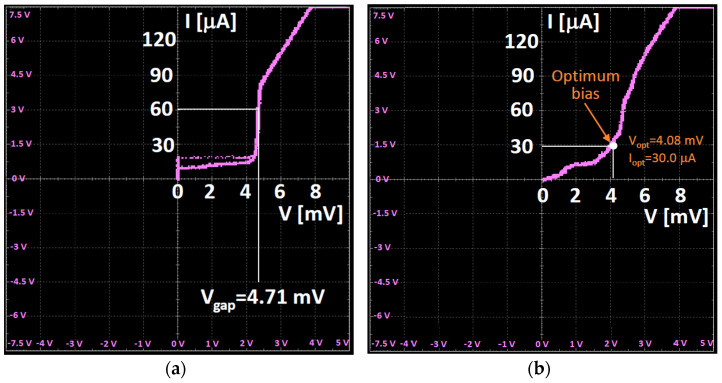
(**a**) I-V characteristic of the SIS mixer without the local oscillator power applied. (**b**) I-V characteristic of the mixer with the local oscillator power applied.

After the evaluation of the output power levels of the local oscillator, the I-V characteristic of the SIS junction of the two-junction array mixer is measured [47]. The measurement is carried out by connecting the digital oscilloscope to the SIS mixer junction bias module of the control system. In Figure 12, the measurement results are shown. In particular, the curve of Figure 12a shows the I-V characteristic of the mixer without the local oscillator power applied, where a spurious Josephson current is present (unsuppressed, as the mixer does not have a magnetic circuit), whereas the curve of Figure 12b represents the case with the local oscillator biasing at 87.5 GHz.

### 5.2. Measurements of the Noise Temperature of the System

When a radio telescope detects a signal, the noise from the receiver is added to it. The most important requirement to be met in a receiver design is the minimisation of the self-produced thermal noise, known as the equivalent noise temperature of the system. A low noise temperature implies the high sensitivity of the receiver [48].

**Figure 13:**
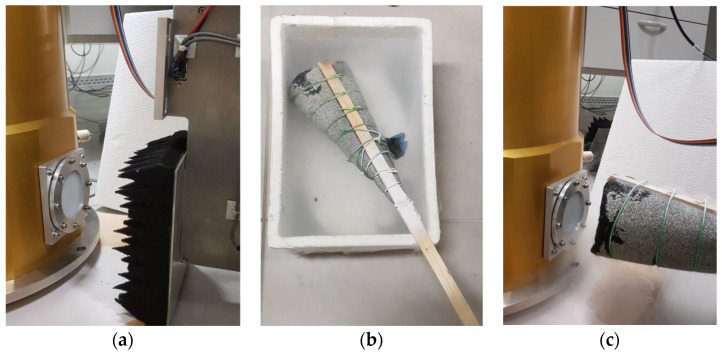
(**a**) Room temperature load placed in front of the HDPE system and the feed of the receiver. (**b**) Absorbing panel immersed in a container filled with liquid nitrogen. (**c**) Cold load placed in front of the HDPE system and the feed of the receiver.

As described in Section 2, a two-load calibration subsystem is included in the original version of the ex-PdBI receiver, which is useful for the calibration when it is installed on the telescope, allowing the measurement of the receiver noise. In our laboratory, we performed noise temperature measurements at several frequencies using the cold and ambient load (Y factor) method [49], without using the internal cryogenic calibration load. Some photos of the experiment are shown in Figure 13. In particular, the absorbing panel (i.e., Emerson & Cuming AN72 material) of the calibration system was used as the room temperature load (see Figure 13a). The cold load was an absorbing panel immersed in a container filled with liquid nitrogen in such a way that the load temperature was lowered to the one of the liquid nitrogen temperature, 77 K (see Figure 13b). The room temperature and cold loads were alternately placed in front of the HDPE vacuum window (and, consequently, in front of the feed of the receiver, as shown in Figure 13c).

A power meter was used as back end and the noise temperature was calculated using the following formulas [49]:

(2) $$Y=\frac{P_{hot}}{P_{cold}}$$

(3) $$T_{rec}=\frac{T_{hot}-Y\cdot T_{cold}}{Y-1}$$

where *Y* represents the ratio between the measured power level *P_hot_* when the hot load is positioned in front of the receiver, and the measured power level *P_cold_* when the cold load is considered. The noise temperature of the receiver *T_rec_* is calculated using formula (3), where for the radiated temperature *T_hot_* we assumed the room temperature (approximately 293 K) and for *T_cold_* the nitrogen temperature of the cold load (approximately 77 K).

**Figure 14:**
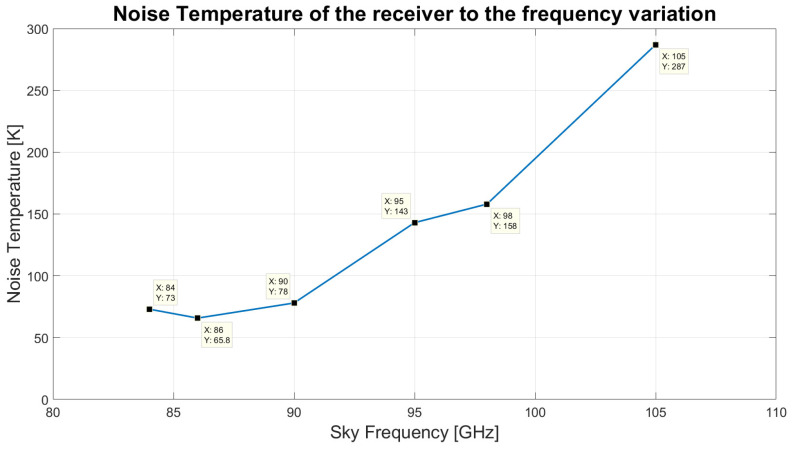
Noise temperature of the upgraded ex-PdBI receiver to the frequency variation.

Table 2 summarises the results of the experiment in the frequency range between 84 and 115 GHz. The measured receiver noise temperature varies considerably as a function of the LO frequency, as shown in the plot of Figure 14. The noise has a minimum of approximately 66 K around 86 GHz RF and increases to approximately 290 K at 105 GHz RF. The measured noise is considerably higher than the one delivered by the original PdBI receiver (where noise of order Trec ≈ 30 K was reported), which was based on a third cryogenic stage cryogenically cooled by liquid helium at 4.2 K, allowing the thermal cooling of the SIS mixer to less than half of the critical temperature of the niobium superconductor (with Tc ≈ 9.2 K). The discrepancy between the receiver noise temperatures measured by IRAM in the original receiver configuration and the one measured at INAF with the modified receiver are mainly due to the different physical temperatures achieved for the third stage (4 K stage), where the mixer is thermalised. The receiver noise is expected to degrade considerably if the physical temperature of the SIS mixer is increased beyond ≈4.5 K. A cryogenic temperature of approximately 6.6 K (much greater than the required ≈4 K value) was achieved during the laboratory tests, in part due to the limited thermal cooling power delivered by the ARS cold head, and in part due to the non-optimal thermal isolation of the coldest cryogenic stage, which requires improvement. This aspect caused a degradation of the receiver noise performance. However, the noise temperature results achieved in the frequency bandwidth 84–95 GHz are considered in line with the initial goals of this project, which aimed at testing the capabilities of the SRT in the 3 mm frequency range. The receiver, if installed on the SRT, could also be suitable to conduct millimetre VLBI experiments at 86 GHz [50].

## 6. Conclusions and Future Work

We presented the feasibility study and preliminary tests for the installation on the SRT of a W-band radio receiver based on a SSB SIS mixer. The instrument was expected to provide very low noise receiver performance across the frequency range 84–116 GHz if the SIS mixer is cryogenically cooled at a physical temperature of approximately 4 K. It was developed by IRAM for the PdBI antennas. A new dedicated electronic control system was designed and developed by our INAF team for its remote control. The feasibility study of the receiver optics, the upgrade of the cooling system, and the evaluation of using a new dedicated local oscillator were presented. The local oscillator system is the same type adopted for the ALMA Band 3 project. The local oscillator was successfully tested and evaluated in the laboratory. The SSB receiver noise temperature measured in the laboratory, at the frequency bandwidth of 84–95 GHz, was less than 150 K, although the temperature of the third cryogenic stage to which the SIS mixer was thermalised was far from optimum, and of approximately 6 K (the recommended temperature of the SIS mixer should be less than approximately 4.5 K). In particular, the noise temperature at 86 GHz was less than 70 K, which would be acceptable for preliminary testing of the SRT active surface, metrology system and evaluating the atmospheric noise contribution and system noise at the 600 m altitude of the SRT site. The receiver could also be used for millimetre VLBI experiments at 86 GHz.

In future work, the receiver could be modified for improved thermal isolation of the third cryogenic stage of the cold head. The receiver noise would considerably improve (decrease) by reducing the physical noise of the third stage.

The experience gained in this work proved to be useful for the recent project to upgrade SRT to allow observations at high radio frequencies. The specific details of this project are described in [27].

## Figures and Tables

**Table 1:** Summary of the technical features and functioning of each subsystem of the new electronic control system.

Module Name	Technical Features	Functioning
Raspberry Pi 2 (Model B)	- 900 MHz quad-core ARM Cortex-A7 CPU; - 1 GB RAM; - Ethernet port; - 4 USB ports.	Core of the new control system.
Arduino UNO REV3	- 16 MHz ATmega328P; - UART, I2C, SPI; - USB port.	Each microcontroller manages one block of the new control system.
Mixer Biasing Block	- I/O Voltage: 5 V, 12 V, 15 V; - 16-bit resolution ADC ADS1115.	Manage the IRAM Junction Bias board for the SIS mixer biasing.
Mixer Tuning and Receiver Calibration Block	- I/O Voltage: 5 V, 12 V, 15 V; - 16-bit resolution ADC ADS1115.	Composed of the SIS mixer backshort motor control board for the mixer tuning, and the receiver calibration motors control board for the receiver calibration.
Monitoring and Control Boards for the ALMA Local Oscillator Block	- I/O Voltage: +6 V @ 1.5 A, ±15 V @ 500 mA; - 16-bit resolution ADC ADS1115; - SPI bus.	Composed of two modules: one for managing the AMC and the other for controlling the PA of the ALMA local oscillator.

**Table 2:** Results of the noise temperature measurements for SIS mixer tuned in LSB at several LO frequencies.

RF Bandwidth (GHz)	Local Oscillator Frequency (GHz)	Voltage and Current Biasing Values	Noise Temperature (K)
83.7–84.3	85.5	V = 4.07 mV; I = 28.0 μA	73
85.7–86.3	87.5	V = 4.08 mV; I = 30.0 μA	65.8
89.7–90.3	91.5	V = 4.06 mV; I = 34.5 μA	78
94.7–95.3	96.5	V = 4.05 mV; I = 31.7 μA	143
98.2–98.8	100	V = 4.03 mV; I = 28.7 μA	158
104.7–105.3	106.5	V = 3.93 mV; I = 28.5 μA	287

## Data Availability

Not applicable.

## References

[1] Nasir F.T., Castiglia C., Buffa F., Deiana G.L., Delitala A., Tarchi A. (2013). Weather forecasting and dynamic scheduling for a modern cm/mm wave radiotelescope. Exp. Astron..

[2] Navarrini A., Olmi L., Nesti R., Buffa F., Orlati A. (2020). Estimates of System Noise Temperature in W-Band at SRT and Effects of Beam Truncation Due to the Gregorian Radome.

[3] IEEE Standard Letter Designations for Radar-Frequency Bands. https://ieeexplore.ieee.org/document/29086.

[4] Ansdell M., Gaidos E., Hedges C., Tazzari M., Kraus A.L., Wyatt M.C., Kennedy G.M., Williams J.P., Mann A.W., Angelo I. (2019). Are inner disc misalignments common? ALMA reveals an isotropic outer disc inclination distribution for young dipper stars. Mon. Not. R. Astron. Soc..

[5] Vigorito A., Calabrese C., Melandri S., Caracciolo A., Mariotti S., Giannetti A., Massardi M., Maris A. (2018). Millimeter-wave spectroscopy and modeling of 1,2-butanediol: Laboratory spectrum in the 59.6–103.6 GHz region and comparison with the ALMA archived observations. Astron. Astrophys..

[6] Biver N., Bockelée-Morvan D., Debout V., Crovisier J., Boissier J., Lis D.C., Russo N.D., Moreno R., Colom P., Paubert G. (2014). Complex organic molecules in comets C/2012 F6 (Lemmon) and C/2013 R1 (Lovejoy): Detection of ethylene glycol and formamide. Astron. Astrophys..

[7] Prestage R.M., Anderson R., Brandt J., Egan D., Lockman F.J., McCullough R., Whitehead M. The Green Bank Telescope: A status update. Proceedings of the 2017 United States National Committee of URSI National Radio Science Meeting (USNC-URSI NRSM).

[8] Green Bank Observatory Website GBT Receivers & Frequency Ranges. https://greenbankobservatory.org/science/gbt-observers/gbt-receivers-and-frequency-ranges/.

[9] Atacama Large Millimeter/Submillimeter Array. https://www.almaobservatory.org/.

[10] (2023). ALMA “ALMA Cycle 10 Technical Handbook”, Doc 10.3, ver. 1.3. https://almascience.nrao.edu/documents-and-tools/cycle10/alma-technical-handbook.

[11] Tan G.H. The ALMA Front Ends; An Overview. Proceedings of the 19th International Symposium on Space Terahertz Technology.

[12] Claude S., Dindo P., Erickson D., Jiang F., Yeung K., Derdall D., Duncan D., Garcia D., Henke D., Leckie B. The Band 3 receiver (84–116 GHz) for ALMA. Proceedings of the 2005 Joint 30th International Conference on Infrared and Millimeter Waves and 13th International Conference on Terahertz Electronics.

[13] Yagoubov P., Mroczkowski T., Belitsky V., Cuadrado-Calle D., Cuttaia F., Fuller G.A., Gallego J.-D., Gonzalez A., Kaneko K., Mena P. (2020). Wideband 67–116 GHz receiver development for ALMA band 2. Astron. Astrophys..

[14] The Institut de Radioastronomie Millimétrique (IRAM) The Plateau de Bure Interferometer (PdBI). https://web-archives.iram.fr/PDBI/.

[15] NOEMA—Twelve Antennas Working Together, Gazing at the Cosmos. https://iram-institute.org/observatories/noema/.

[16] Chenu J.Y., Navarrini A., Bortolotti Y., Butin G., Fontana A.L., Mahieu S., Maier D., Mattiocco F., Serres P., Berton M. (2016). The Front-End of the NOEMA Interferometer. IEEE Trans. Terahertz Sci. Technol..

[17] INAF—Istituto di Radioastronomina—INAF Radiotelescopes. https://info.ira.inaf.it/en/.

[18] Ladu A., Schirru L., Gaudiomonte F., Marongiu P., Angius G., Perini F., Vargiu G.P. (2022). Upgrading of the L-P Band Cryogenic Receiver of the Sardinia Radio Telescope: A Feasibility Study. Sensors.

[19] Peverini O.A., Tascone R., Virone G., Addamo G., Olivieri A., Orta R. C-band dual-polarization receiver for the Sardinia Radio-Telescope. Proceedings of the 2009 International Conference on Electromagnetics in Advanced Applications.

[20] Orfei A., Carbonaro L., Cattani A., Cremonini A., Cresci L., Fiocchi F., Maccaferri A., Maccaferri G., Mariotti S., Monari J. (2010). A Multi-Feed Receiver in the 18 to 26.5 GHz Band for Radio Astronomy. IEEE Antennas Propag. Mag..

[21] Navarrini A., Orfei A., Nesti R., Valente G., Mariotti S., Bolli P., Pisanu T., Roda J., Cresci L., Marongiu P. Front-ends and phased array feeds for the Sardinia Radio Telescope. Proceedings of the 32nd URSI GASS.

[22] Egron E., Pellizzoni A., Giroletti M., Righini S., Stagni M., Orlati A., Migoni C., Melis A., Concu R., Barbas L. (2017). Single-dish and VLBI observations of Cygnus X-3 during the 2016 giant flare episode. Mon. Not. R. Astron. Soc..

[23] Pilia M., Burgay M., Possenti A., Ridolfi A., Gajjar V., Corongiu A., Perrodin D., Bernardi G., Naldi G., Pupillo G. (2020). The lowest-frequency fast radio bursts: Sardinia radio telescope detection of the periodic FRB 180916 at 328 MHz. Astrophys. J..

[24] Schirru L., Pisanu T., Podda A. (2021). The Ad Hoc Back-End of the BIRALET Radar to Measure Slant-Range and Doppler Shift of Resident Space Objects. Electronics.

[25] Schirru L., Pisanu T., Navarrini A., Urru E., Gaudiomonte F., Ortu P., Montisci G. Advantages of Using a C-band Phased Array Feed as a Receiver in the Sardinia Radio Telescope for Space Debris Monitoring. Proceedings of the 2019 IEEE 2nd Ukraine Conference on Electrical and Computer Engineering (UKRCON).

[26] Bolli P., Olmi L., Roda J., Zacchiroli G. (2014). A novel application of the active surface of the shaped Sardinia radio telescope for primary-focus operations. IEEE Antennas Wirel. Propag. Lett..

[27] Govoni F., Bolli P., Buffa F., Caito L., Carretti E., Comoretto G., Fierro D., Melis A., Murgia M., Navarrini A. The high-frequency upgrade of the Sardinia Radio Telescope. Proceedings of the 2021 XXXIVth General Assembly and Scientific Symposium of the International Union of Radio Science (URSI GASS).

[28] Blondel J., Carter M., Karpov A., Lazareff B., Mattiocco F., Lamb J. (1996). Dual-Channel SIS Receivers for the IRAM Plateau de Bure Interferometer. Int. J. Infrared Millim. Waves.

[29] Guilloteau S., Delannoy J., Downes D., Greve A., Guelin M., Lucas R. (1992). The IRAM interferometer on Plateau de Bure. Astron. Astrophys..

[30] Ladu A., Pisanu T., Navarrini A., Marongiu P., Valente G. A 3mm band SIS receiver for the Sardinia Radio Telescope. Proceedings of the SPIE Astronomical Telescopes + Instrumentation.

[31] Ladu A., Ortu P., Saba A., Pili M., Guadiomonte F., Navarrini A., Urru E., Pisanu T., Valente G., Marongiu P. The control system of the 3 mm band SIS receiver for the Sardinia Radio Telescope. Proceedings of the SPIE Astronomical Telescopes + Instrumentation.

[32] Navarrini A., Fontana A.L., Maier D., Serres P., Billon-Pierron D. (2016). Superconductor-Insulator-Superconductor Mixers for the 2 mm Band (129–174 GHz). J. Infrared Millim. Terahertz Waves.

[33] Navarrini A., Lazareff B. Design of 129–174 GHz SSB SIS mixer for Band 2 of New Generation Receiver of IRAM PdB Interferometer. Proceedings of the 14th International Symposium on Space Terahertz Technology.

[34] The Institut de Radioastronomie Millimétrique (IRAM) Local Oscillator System. https://web-archives.iram.fr/TA/receiver/rxtutorial/losyst.htm.

[35] Gallego J.D., Pospieszalski M.W. Design and performance of cryogenically-coolable ultra-low noise, L-band amplifier. Proceedings of the 20th European Microwave Conference.

[36] Infrared Laboratories Liquid Cryogen Dewars. https://www.irlabs.com/products/cryostats/liquid-cryogen-dewars/.

[37] CTI-Cryogenics Installation, Operation and Servicing Instructions. Model 350C Cryodyne Cryocooler. http://docs.eao.hawaii.edu/JCMT/a/024_helium_compressors/06/CTI%20350CwithSC_Compressor-1985.pdf.

[38] Emerson&Cuming Anechoic Chambers. https://www.ecanechoicchambers.com/.

[39] Bryerton E., Saini K., Morgan M., Stogoski M., Boyd T., Thacker D. Development of Electronically Tuned Local Oscillators for ALMA. Proceedings of the 30th Conference on Infrared and Millimeter Waves & 13th International Conference on Terahertz Electron.

[40] Rohde and Schwarz R&S^®^SMF100A Microwave Signal Generator. https://www.rohde-schwarz.com/it/prodotti/misura-e-collaudo/generatori-di-segnali-analogici/rs-smf100a_63493-8447.html.

[41] Advanced Research Systems: Complete Cryogenic Solutions for Your Experimental Application from 1.5 K to 800 K. https://www.arscryo.com/.

[42] Raspberry Pi 2 Model B. https://www.raspberrypi.org/products/raspberry-pi-2-model-b/.

[43] Arduino Uno Rev3 SMD. https://store.arduino.cc/products/arduino-uno-rev3-smd.

[44] ADS1115 16-Bit ADC—4 Channel with Programmable Gain Amplifier—STEMMA QT/Qwiic. https://www.adafruit.com/product/1085.

[45] Tucker J.R., Feldman M.J. (1985). Quantum detection at millimeter wavelengths. Rev. Mod. Phys..

[46] VDI: Erikson Power Meters (PM5). https://www.vadiodes.com/en/products/power-meters-erickson.

[47] Photon-Assisted Tunnelling. https://web-archives.iram.fr/TA/receiver/rxtutorial/photonass.htm.

[48] Wang K., Chen M.Z., Ma J., Cao L., Yan H., Li X., Xiang B. (2016). Aerating System of Keeping Dry for Vacuum Window of Cryogenic Receiver. Hydraul. Pneum..

[49] Wang K., Wang Y., Chen M.Z., Li X.F., Ma J., Yan H., Xiang B.B. (2019). Noise Temperature Measurement System of Normal and High Temperature Load Method. J. Phys. Conf. Ser..

[50] Akiyama K., Kauffmann J., Matthews L.D., Moriyama K., Koyama S., Hada K. (2023). Millimeter/Submillimeter VLBI with a Next Generation Large Radio Telescope in the Atacama Desert. Galaxies.

